# Tibolone inhibits bone resorption without secondary positive effects on cartilage degradation

**DOI:** 10.1186/1471-2474-9-153

**Published:** 2008-11-18

**Authors:** MA Karsdal, I Byrjalsen, DJ Leeming, C Christiansen

**Affiliations:** 1 Nordic Bioscience A/S, Herlev, Denmark

## Abstract

**Background:**

Osteoarthritis is associated with increased bone resorption and increased cartilage degradation in the subchondral bone and joint. The objective of the present study was to determine whether Tibolone, a synthetic steroid with estrogenic, androgenic, and progestogenic properties, would have similar dual actions on both bone and cartilage turnover, as reported previously with some SERMS and HRT.

**Methods:**

This study was a secondary analysis of ninety-one healthy postmenopausal women aged 52–75 yrs entered a 2-yr double blind, randomized, placebo-controlled study of treatment with either 1.25 mg/day (n = 36), or 2.5 mg/day Tibolone (n = 35), or placebo (n = 20), (J Clin Endocrinol Metab. 1996 Jul;81(7):2419–22) Second void morning urine samples were collected at baseline, and at 3, 6, 12, and 24 months. Urine CrossLaps^® ^ELISA (CTX-I) and Urine CartiLaps^® ^ELISA (CTX-II) was investigated as markers of bone resorption and cartilage degradation, respectively.

**Results:**

Tibolone significantly (P < 0.001) suppressed bone resorption by approximately 60%. In contrast, no effect was observed on cartilage degradation.

**Conclusion:**

These data suggest uncoupling of the bone and cartilage effects of the synthetic steroid, Tibolone. Bone resorption was significantly decreased, whereas cartilage degradation was unchanged. These effects are in contrast to those observed some SERMs with effects on both bone and cartilage degradation. These effects may in part be described by the complicated pharmacology of Tibolone on testosterone, estrogen and progesterone receptors.

## Background

Osteoarthritis (OA) is the most common form of arthritis [1]. One hallmark of the disease is progressive degeneration of articular cartilage, generation of osteophytes and subsequent joint space narrowing. This progression of disease may involve both bone and cartilage parameters, which in some instances may be tightly coupled.

The relationship between bone and cartilage degradation in OA is a complex. A apparent co-existence between the two processes exists [2,3], although the cellular and molecular mechanism remains to be further investigated and identified [4]. Several groups have demonstrated an accelerated incidence of OA in women following menopause [5,6] which in part may be caused by increased bone resorption [7,8]. Studies investigating gender and age as risk factors of developing OA indicate that OA increases with age and women are at a higher risk than men [9]., which supports the notion that sex hormones are related to the incidence of OA. In addition, pre-clinical studies in monkeys [10] and rats [11] have shown that estrogen depletion results in increased bone turnover, leading to altered subchondral bone structure and decreased articular cartilage integrity.

In clinical settings a number of studies have provided evidence for the coupling between bone and cartilage degradation. A selective estrogen-receptor modulator (SERM) was shown to protect against both bone and cartilage degradation, by restoring turnover of both compartments to pre-menopausal level during a 12 month period [12]. In similar context, cartilage degradation was assessed in 384 postmenopausal women, and was found to be significantly lower in women using hormone replacement therapy (HRT) compared to control [6,12], and the cartilage degradation was significantly higher in postmenopausal women when compared to an age-matched group of pre-menopausal women [6].

Tibolone is a synthetic steroid [(7-α,l7-α & 17-hydroxy-7-methyl-l9-norpregn-5(1O)-en-20-yn-3-one, Org OD 14, Livial, Organon, The Netherlands] with a combination of estrogenic, androgenic, and progestogenic properties, is capable of relieving climacteric symptoms [13-15] with almost no stimulatory effect on the endometrium [16]. Tibolone is metabolized in the intestine and liver into 3 active metabolites: 3α- and 3β-hydroxy metabolites and Δ^4^-7alpha-methylnorethisterone. The first two metabolites bind to estrogen receptors, primarily ERα receptors, and have estrogenic effects on bone, thermoregulatory centers in the central nervous system, and the vagina [17-19]. The Δ4-7alpha-methylnorethisterone metabolite binds to the progesterone and androgen receptors. Tibolone is primarily used to treat women with climacteric complaints for whom bleeding is unacceptable, or who have experienced side-effects during conventional HRT. The estrogenic characteristics of tibolone imply an estrogen-like effect on bone as well, which have been documented and characterized previously [20,21].

Bone and cartilage degradation can be assed by biochemical markers. Bone resorption by osteoclasts is for the major part mediated by the protease cathespin K, which results in the specific degradation fragment of collagen type I, CTX-I [22,23]. CTX-I fragments have been used as a surrogate measure of bone resorption for *in vitro*, preclinical and clinical studies [22,24]. With respect to cartilage degradation, CTX-II, is a matrix metalloprotease (MMP) generated fragment of collagen type II which is predominately found in articular cartilage [25,26]. CTX-II levels has been shown to predict structural progression of osteoarthritis under several clinical settings [27].

As several lines of evidence suggest a tight coupling between bone and cartilage turnover [28], and protective effects of estrogens and some SERMs [28], the objective of the present study was to determine whether Tibolone, a synthetic steroid with estrogenic, androgenic, and progestogenic properties, would have similar dual actions on both bone and cartilage turnover.

## Methods

This study is a secondary analysis of the effects published previously [21]. Ninety-one healthy postmenopausal women aged 52–75 yrs entered a 2-yr double blind, randomized, placebo-controlled study of treatment with either 1.25 mg/day (n = 36), or 2.5 mg/day tibolone (n = 35), or placebo (n = 20) as previously described [21]. Second void morning urine samples were collected at baseline, and at 3, 6, 12, and 24 months. The biochemical marker CTX-II was measured in urine by Urine CartiLaps^® ^ELISA (Nordic Bioscience A/S) as marker of cartilage degradation.

As described in the original publication, [21], after being introduced thoroughly about the trial, participants gave their informed consent to participate (Helsinki Declaration II). The study was approved by the ethical committee of Copenhagen County, Denmark.

### Statistical analysis

To assess longitudinal changes, the values were calculated for each person and expressed as the percentage of the initial baseline value. The data of the creatinine-corrected values of CTXI and CTXII, and the relative changes were logarithmically transformed to obtain normality and symmetry of variances. The trapezoidal method was applied for calculation of the time-averaged mean change from baseline. Analysis of variance (ANOVA) was used for comparison of baseline data between treatment groups. The two-tailed Student's *t*-test was applied for pairwise comparison of data from the active treatment groups with placebo.

For all tests *p *< 0.05 was considered significant. All statistical calculations were performed using the SAS software package (release 9.1, SAS Institute Inc., Cary, NC, USA).

## Results

Table 1 shows the baseline demographic data of the participants within each treatment group who completed the 2-year study protocol as published previously [21]. The group receiving 2.5 mg of tibolone was of slightly higher height (p = 0.04). In all other aspects of age, weight, BMI, years since menopause, and the biochemical markers of urinary CTX-I and CTX-II there were no significant differences among the groups.

**Table 1:** Values shown are mean (SD) or ^a^geometric mean (± 1 SD range)

	**Placebo** **(n = 13)**	**1.25 mg** **(n = 29)**	**2.5 mg** **(n = 28)**
Age (yrs)	68.3 (6.0)	66.5 (7.0)	64.0 (6.7)
YSM (yrs)	20 (6.2)	19.6 (6.1)	17.5 (6.5)
Height (cm)	158.5 (5.3)	159.7 (5.9)	162.9 (6.1)
Weight (kg)	61.8 (6.8)	62.5 (9.5)	61.1 (6.8)
BMI (kg/m^2^)	24.7 (3.2)	24.5 (3.4)	23.1 (2.8)
^a^CTXI (μg/mmol)	0.26 (0.18–0.37)	0.32 (0.21–0.49)	0.31 (0.22–0.45)
^a^CTXII (μg/mmol)	0.13 (0.08–0.26)	0.17 (0.09–0.32)	0.13 (0.07–0.24)

### The effect of tibolone on bone resorption

**Figure 1:**
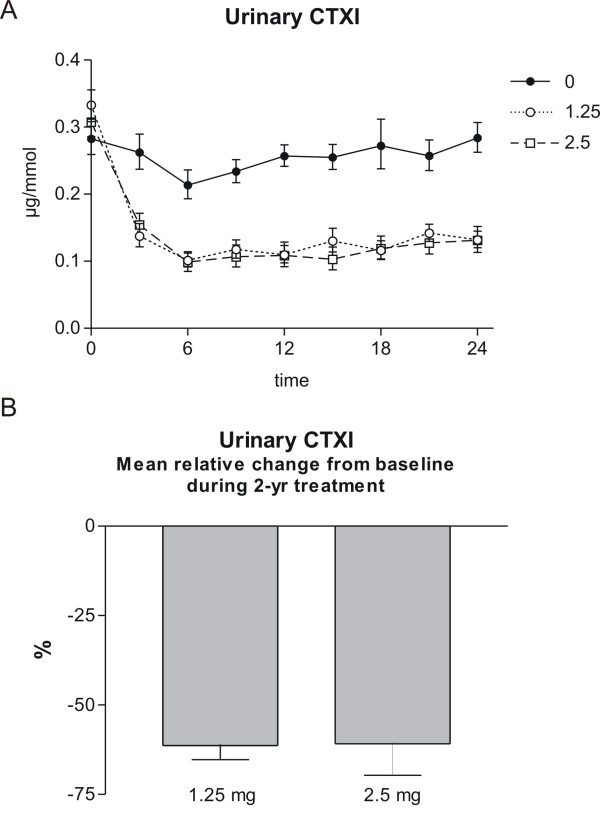
**Bone resorption. Upper panel: Urinary CTXI during 2 years of therapy in the groups receiving 2.5 mg, 1.25 mg of tibolone, or placebo**. Values shown are geometric mean ± 1 SEM. B. Lower panel: Mean relative change from baseline in urinary CTXI during 2 years of therapy with 1.25 and 2.5 mg of tibolone. Values shown are placebo-corrected time-averaged mean change during the treatment period and given as mean ± 1 SEM. The level of significance denotes difference from the placebo group: *** p < 0.001. Modified with permission from [21].

Bone resorption was investigated by measurement of urinary CTX-I during the 2 years of therapy every 3^rd ^month in the groups receiving 2.5 mg or 1.25 mg of tibolone, or placebo. Tibolone inhibited bone resorption highly significant with a plateau after 6, as seen in figure 1, upper panel. The relative change from baseline in urinary CTX-I during 2 years of therapy with 1.25 and 2.5 mg of tibolone is presented in figure 1, lower panel. Values shown are placebo-corrected time-averaged mean change during the treatment period and given as mean ± 1 SEM. The level of significance denotes difference from the placebo group: *** p < 0.001. Data are modified with permission from [21].

### The effect of tibolone on cartilage degradation

**Figure 2:**
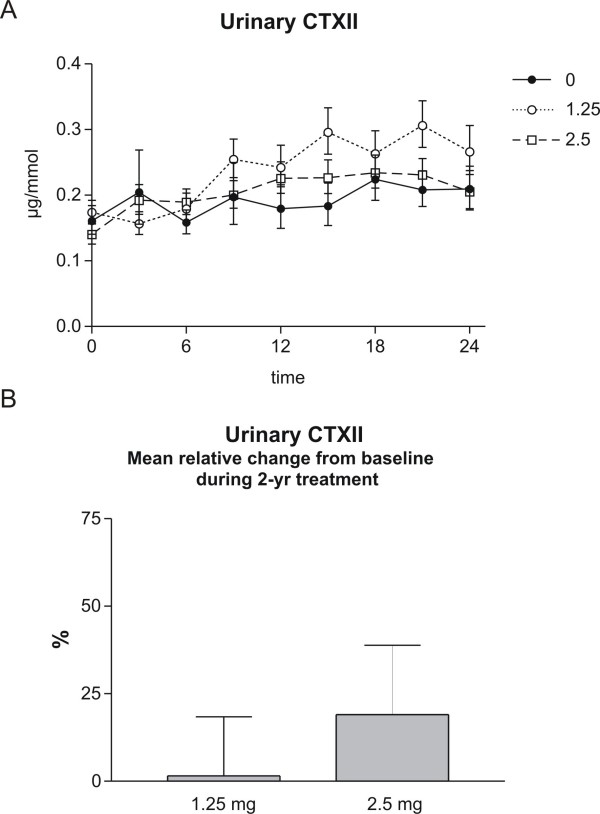
**Cartilage degradation: Upper panel: Urinary CTXII (lower panel) during 2 years of therapy in the groups receiving 2.5 mg, 1.25 mg of tibolone, or placebo**. Values shown are geometric mean ± 1 SEM. Lower panel: Mean relative change from baseline in urinary CTXII during 2 years of therapy with 1.25 and 2.5 mg of tibolone. Values shown are placebo-corrected time-averaged mean change during the treatment period and given as mean ± 1 SEM. Levels did not reach significance.

Cartilage degradation was measured by measurement of urinary CTX-II during the 2 years of therapy every 3^rd ^month in the groups receiving 2.5 mg or 1.25 mg of tibolone, or placebo. Tibolone did not affect cartilage degradation, as presented in figure 2, upper panel. The relative change from baseline in urinary CTXII during 2 years of therapy with 1.25 and 2.5 mg of tibolone is presented in figure 2, lower panel. Values shown are placebo-corrected time-averaged mean change during the treatment period and given as mean ± 1 SEM.

## Discussion

Experimental and clinical observations suggest that the structural integrity of articular cartilage is dependent on normal subchondral bone turnover, intact chondrocyte function and ordinary biomechanical stresses [3,29-32]. Because there is an apparent inter-relationship between the subchondral bone and the articular cartilage, interventions with effect on bone turnover may possibly have secondary or direct effects on cartilage turnover.

The presented data suggest that bone resorption can be strongly attenuated without the secondary positive effects on cartilage degradation. In fact, a trend toward increased cartilage degradation that may have become statistical significant if more samples had been available was observed. These data indicate that the tight coupling between bone and cartilage metabolism can be disassociated under some circumstances. This uncoupling is an important difference compared to that other estrogen like molecules and SERMS that have displayed protective effects on both bone and cartilage degradation. In the present study the observed effect size of 2.5 mg of Tibolone was an increase of 19% [95% confidence interval -17%;+70%] in CTXII. This is in contrast to other interventions that all have shown a decrease in CTXII, e.g. risedronate a decrease of 30% [33,34], estrogen replacement therapy a decrease of 25% [35], strontium ranelate a decrease of 15–20% [36] and levormeloxifene with a decrease of 50% [12].

### Bone and OA

An increasing amount of attention has been devoted to the role of the bone in the pathogenesis of osteoarthritis. Subchondral bone sclerosis, alterations in the trabecular structure, lesions (previously called development of bone marrow edema) and osteophytes are important features of the pathology of OA [2,10,29,37-39]. The present understanding remains on an observational level, in which an increasing range of experimental evidence is emerging. In support of the important role of bone turnover in the pathogenesis of OA, several independent lines of experimental evidence are found [3,29-32]. In brief, examinations of peri-articular bone in knees and hips with OA have confirmed that the subchondral bone is abnormal in OA joints, which altered trabecular structure, sclerosis of the subchondral plate [40], as well increased bone turnover [41,42]. Cross-sectional studies have also established that women with advanced knee or hip OA have a higher bone mineral density (BMD) near or at the site of joint OA [43]. Plausible proof of a link between bone and cartilage recently came from an animal model of OA, where extensive inhibition of bone resorption resulted in a 50% decrease in cartilage pathology score assessed by Mankin score [29,38]. In addition, accelerated bone turnover has in both traumatic and estrogen deficiency models (ovariectomy (OVX)) been shown to augment articular cartilage erosion [7,11,12,44,45], in which increased bone resorption alone results in increased articular cartilage damage [10,37,46].

Thereby, interventions that may positively affect bone turnover, may in theory have secondary positive effects on progression of OA. The present data strongly suggest that these processes can be uncoupled. This might in part be mediated through the androgenic properties of tibolone, as androgens in contrast to that of estrogens might have deleterious effects on cartilage health [45]. However the effects of androgens and the direct on articular cartilage compared to that of estrogens needs additional attention.

### Estrogen and OA

The putative positive of some estrogens of cartilage health may both be though indirect and direct effects on cartilage. Estrogens are anti-resorptives that directly and indirectly attenuates osteoclastogenesis and osteoclastic resorption [47]. At the same time, chondrocytes express estrogen receptors, and respond to estrogen [11,48-52].

The positive long-term beneficial effect of estrogen for prevention of OA was recently demonstrated in 180 female cynomolgus monkeys [10], receiving estrogen replacement therapy (ERT) treatment for 3 years. Significant less cartilage lesions of OA were seen in the ERT group compared to the control group. In addition, several preclinical models have provided evidence for protective effects of estrogen [11,12,46].

In clinical settings a number of studies have suggested positive effects on cartilage degradation. A SERM was shown to protect against both bone and cartilage degradation [53], and cartilage degradation and was found to be significantly lower in women using hormone replacement therapy (HRT) compared to control [6,12,54], and the cartilage degradation was significantly higher in postmenopausal women when compared to an age-matched group of pre-menopausal women. These data are in alignment with the recent analysis from the WHI studies, which documented that women taking estrogen had 45% less total joint surgery compared to of placebo [55].

However in the present study Tibolone did not result in secondary positive effects on cartilage degradation. This may in part be described by the complicated pharmacology of Tibolone on testosterone, estrogen and progesterone receptors. This is in contrast to others SERM and estrogen like molecules that to a much larger extent is selective for the estrogen receptor.

### Limitations of the current study

The present study has some limitations. Cartilage degradation was evaluated by the biochemical markers u-CTX-II. Levels of u-CTX-II have been shown to correlate with cartilage damage in both animal models and clinical trials [7,11,27,56], however long term randomized clinical trials studies are needed to further investigate and document the putative positive effects of estrogens on the pathogenesis of osteoarthritis.

Biochemical markers of cartilage degradation measured in the systemic fluids, serum and urine, are the net results of the biological activity of all joints and tissues in which collagen type II present. CTX-II is generated by MMP activity [57] have been shown to be produced by catabolically stimulated articular cartilage, and present in damaged articular cartilage [25,56]. However, CTX-II may in addition be generated by the cartilage of non-synovial joints. The main contributors of cartilage degradation biomarkers have been shown to be; knees, hips, hands, vertebral facet joints in addition to spinal disc degeneration (DD) [58,59]. In addition, a smaller contribution of CTX-II may originate from the calcified cartilage in the subchondral bone area [28]. The contribution of each cartilage compartment to the total pool of cartilage degradation measured is important to further understand, for the interpretation of the effect on the total pool of any biochemical marker. Some insights into the relative contribution of the different joints to the total amount of CTX-II have been provided [58]. CTX-II was shown to be related to the number of joint affected, evaluated by radiological OA, in which generalised resulted in an approximately 100% increase in CTX-II [58]. In addition, CTX-II was very recently shown to predict medial knee articular cartilage loss evaluated by quantitative MRI [60]. With respect to the current study, each compartment may have contributed differently to the pool of CTX-II, also in response to therapy. Further research is needed to understand the effects of estrogen and estrogen related compounds on the individual joints.

The analysis was performed by re-analysis on stored samples, which to some extend may have influenced that measurements. However, in house data suggest that u-CTX-II is stable for more than 3 years under the appropriate conditions.

## Conclusion

In summary, we describe for the first time that anti-resorptive treatments can result in an uncoupling of the bone and cartilage protective effects, measured by biochemical markers and the limitation associated with those. These findings may result in questioning of the coupling between bone and cartilage, and whether they indeed are separate or coupled processes.

## Competing interests

All authors are full time employees of Nordic Bioscience, a company engaged in the development of biochemical markers of bone and cartilage turnover. Morten a. Karsdal and Claus Christiansen hold stocks in Nordic Bioscience.

## Authors' contributions

CC participated in designing the original study and reviewed the last version of the manuscript. IB performed statistical analysis and participated in drafting of the manuscript. DJL participated in data analysis and writing of the manuscript. MAK came up with the original idea for the study, drafted the first manuscript and finalized the last version of the manuscript.

## Pre-publication history

The pre-publication history for this paper can be accessed here:



## References

[B1] Abramson SB, Attur M, Yazici Y (2006). Prospects for disease modification in osteoarthritis. Nat Clin Pract Rheumatol.

[B2] Carbone LD, Nevitt MC, Wildy K, Barrow KD, Harris F, Felson D (2004). The relationship of antiresorptive drug use to structural findings and symptoms of knee osteoarthritis. Arthritis Rheum.

[B3] Felson DT, Neogi T (2004). Osteoarthritis: is it a disease of cartilage or of bone?. Arthritis Rheum.

[B4] Felson DT, Nevitt MC (1998). The effects of estrogen on osteoarthritis. Curr Opin Rheumatol.

[B5] Oliveria SA, Felson DT, Klein RA, Reed JI, Walker AM (1996). Estrogen replacement therapy and the development of osteoarthritis. Epidemiology.

[B6] Mouritzen U, Christgau S, Lehmann HJ, Tanko LB, Christiansen C (2003). Cartilage turnover assessed with a newly developed assay measuring collagen type II degradation products: influence of age, sex, menopause, hormone replacement therapy, and body mass index. Ann Rheum Dis.

[B7] Sondergaard BC, Ostergaard S, Christiansen C, Karsdal MA (2007). The Effect of Oral Calcitonin on Cartilage Turnover and Surface Erosion in the Ovariectomized Rat Model.. Arthritis Rheum.

[B8] Sowers MR, McConnell D, Jannausch M, Buyuktur AG, Hochberg M, Jamadar DA (2006). Estradiol and its metabolites and their association with knee osteoarthritis. Arthritis Rheum.

[B9] Felson DT, Zhang Y, Hannan MT, Naimark A, Weissman BN, Aliabadi P (1995). The incidence and natural history of knee osteoarthritis in the elderly. The Framingham Osteoarthritis Study. Arthritis Rheum.

[B10] Ham KD, Loeser RF, Lindgren BR, Carlson CS (2002). Effects of long-term estrogen replacement therapy on osteoarthritis severity in cynomolgus monkeys. Arthritis Rheum.

[B11] Oestergaard S, Sondergaard BC, Hoegh-Andersen P, Henriksen K, Qvist P, Christiansen C (2006). Effects of ovariectomy and estrogen therapy on type II collagen degradation and structural integrity of articular cartilage in rats: implications of the time of initiation. Arthritis Rheum.

[B12] Christgau S, Tanko LB, Cloos PA, Mouritzen U, Christiansen C, Delaisse JM (2004). Suppression of elevated cartilage turnover in postmenopausal women and in ovariectomized rats by estrogen and a selective estrogen-receptor modulator (SERM). Menopause.

[B13] Crona N, Samsioe G, Lindberg UB, Silfverstolpe G (1988). Treatment of climacteric complaints with Org OD 14: a comparative study with oestradiol valerate and placebo. Maturitas.

[B14] Kicovic PM, Cortes-Prieto J, Luisi M, Milojevic S, Franchi F (1982). Placebo-controlled cross-over study of effects of Org OD 14 in menopausal women. Reproduccion.

[B15] Punnonen R, Liukko P, Cortes-Prieto J, Eydam F, Milojevic S, Trevoux R (1984). Multicentre study of effects of Org OD 14 on endometrium, vaginal cytology and cervical mucus in post-menopausal and oophorectomized women. Maturitas.

[B16] Genazzani AR, Benedek-Jaszmann LJ, Hart DM, Andolsek L, Kicovic PM, Tax L (1991). Org OD 14 and the endometrium. Maturitas.

[B17] de Gooyer ME, Deckers GH, Schoonen WG, Verheul HA, Kloosterboer HJ (2003). Receptor profiling and endocrine interactions of tibolone. Steroids.

[B18] Jelinek J, Kappen A, Schonbaum E, Lomax P (1984). A primate model of human postmenopausal hot flushes. J Clin Endocrinol Metab.

[B19] Landgren MB, Helmond FA, Engelen S (2005). Tibolone relieves climacteric symptoms in highly symptomatic women with at least seven hot flushes and sweats per day. Maturitas.

[B20] Geusens P, Dequeker J, Gielen J, Schot LP (1991). Non-linear increase in vertebral density induced by a synthetic steroid (Org OD 14) in women with established osteoporosis. Maturitas.

[B21] Bjarnason NH, Bjarnason K, Haarbo J, Rosenquist C, Christiansen C (1996). Tibolone: prevention of bone loss in late postmenopausal women. J Clin Endocrinol Metab.

[B22] Schaller S, Henriksen K, Hoegh-Andersen P, Sondergaard BC, Sumer EU, Tanko LB (2005). In vitro, ex vivo, and in vivo methodological approaches for studying therapeutic targets of osteoporosis and degenerative joint diseases: how biomarkers can assist?. Assay Drug Dev Technol.

[B23] Schaller S, Henriksen K, Sveigaard C, Heegaard AM, Helix N, Stahlhut M (2004). The chloride channel inhibitor n53736 prevents bone resorption in ovariectomized rats without changing bone formation. J Bone Miner Res.

[B24] Ravn P, Hosking D, Thompson D, Cizza G, Wasnich RD, McClung M (1999). Monitoring of alendronate treatment and prediction of effect on bone mass by biochemical markers in the early postmenopausal intervention cohort study. J Clin Endocrinol Metab.

[B25] Sondergaard BC, Henriksen K, Wulf H, Oestergaard S, Schurigt U, Brauer R (2006). Relative contribution of matrix metalloprotease and cysteine protease activities to cytokine-stimulated articular cartilage degradation. Osteoarthritis Cartilage.

[B26] Nielsen Rh, Stoop R, Leeming DJ, Stolina M, Qvist P, Christiansen C (2008). Evaluation of cartilage damage by measuring collagen degradation products in joint extracts in a traumatic model of osteoarthritis. Biomarkers.

[B27] Reijman M, Hazes JM, Bierma-Zeinstra SM, Koes BW, Christgau S, Christiansen C (2004). A new marker for osteoarthritis: cross-sectional and longitudinal approach. Arthritis Rheum.

[B28] Karsdal MA, Leeming DJ, Dam EB, Henriksen K, Alexandersen P, Pastoureau P (2008). Should subchondral bone turnover be targeted when treating osteoarthritis?. Osteoarthritis Cartilage.

[B29] Hayami T, Pickarski M, Wesolowski GA, McLane J, Bone A, Destefano J (2004). The role of subchondral bone remodeling in osteoarthritis: reduction of cartilage degeneration and prevention of osteophyte formation by alendronate in the rat anterior cruciate ligament transection model. Arthritis Rheum.

[B30] Mansell JP, Collins C, Bailey AJ (2007). Bone, not cartilage, should be the major focus in osteoarthritis. Nat Clin Pract Rheumatol.

[B31] Mansell JP, Bailey AJ (1998). Abnormal cancellous bone collagen metabolism in osteoarthritis. J Clin Invest.

[B32] Bailey AJ, Mansell JP (1997). Do subchondral bone changes exacerbate or precede articular cartilage destruction in osteoarthritis of the elderly?. Gerontology.

[B33] Bingham CO, Buckland-Wright JC, Garnero P, Cohen SB, Dougados M, Adami S (2006). Risedronate decreases biochemical markers of cartilage degradation but does not decrease symptoms or slow radiographic progression in patients with medial compartment osteoarthritis of the knee: results of the two-year multinational knee osteoarthritis structural arthritis study. Arthritis Rheum.

[B34] Garnero P, Bingham C, Aronstein W, Cohen S, Conaghan P, Cline G (2005). Treatment with risedronate reduced urinary CTX-II, a specificbiochemical marker of cartilage type II collagen degradation in a 24 month study of knee OA. ACR Abstract book.

[B35] Ravn P, Warming L, Christgau S, Christiansen C (2004). The effect on cartilage of different forms of application of postmenopausal estrogen therapy: comparison of oral and transdermal therapy. Bone.

[B36] Alexandersen P, Karsdal MA, Qvist P, Reginster JY, Christiansen C (2007). Strontium ranelate reduces the urinary level of cartilage degradation biomarker CTX-II in postmenopausal women. Bone.

[B37] Ham KD, Carlson CS (2004). Effects of estrogen replacement therapy on bone turnover in subchondral bone and epiphyseal metaphyseal cancellous bone of ovariectomized cynomolgus monkeys. J Bone Miner Res.

[B38] Hayami T, Pickarski M, Zhuo Y, Wesolowski GA, Rodan GA, Duong LT (2005). Characterization of articular cartilage and subchondral bone changes in the rat anterior cruciate ligament transection and meniscectomized models of osteoarthritis. Bone.

[B39] Garnero P, Peterfy C, Zaim S, Schoenharting M (2005). Bone marrow abnormalities on magnetic resonance imaging are associated with type II collagen degradation in knee osteoarthritis: a three-month longitudinal study. Arthritis Rheum.

[B40] Hunter DJ, Hart D, Snieder H, Bettica P, Swaminathan R, Spector TD (2003). Evidence of altered bone turnover, vitamin D and calcium regulation with knee osteoarthritis in female twins. Rheumatology (Oxford).

[B41] Hunter DJ, Spector TD (2003). The role of bone metabolism in osteoarthritis. Curr Rheumatol Rep.

[B42] Dieppe P, Cushnaghan J, Young P, Kirwan J (1993). Prediction of the progression of joint space narrowing in osteoarthritis of the knee by bone scintigraphy. Ann Rheum Dis.

[B43] Arden N, Nevitt MC (2006). Osteoarthritis: epidemiology. Best Pract Res Clin Rheumatol.

[B44] Calvo E, Castaneda S, Largo R, Fernandez-Valle ME, Rodriguez-Salvanes F, Herrero-Beaumont G (2006). Osteoporosis increases the severity of cartilage damage in an experimental model of osteoarthritis in rabbits. Osteoarthritis Cartilage.

[B45] Ma HL, Blanchet TJ, Peluso D, Hopkins B, Morris EA, Glasson SS (2007). Osteoarthritis severity is sex dependent in a surgical mouse model. Osteoarthritis Cartilage.

[B46] Hoegh-Andersen P, Tanko LB, Andersen TL, Lundberg CV, Mo JA, Heegaard AM (2004). Ovariectomized rats as a model of postmenopausal osteoarthritis: validation and application. Arthritis Res Ther.

[B47] Sorensen MG, Henriksen K, Dziegiel MH, Tanko LB, Karsdal MA (2006). Estrogen directly attenuates human osteoclastogenesis, but has no effect on resorption by mature osteoclasts. DNA Cell Biol.

[B48] Richette P, Corvol M, Bardin T (2003). Estrogens, cartilage, and osteoarthritis. Joint Bone Spine.

[B49] Dayani N, Corvol MT, Robel P, Eychenne B, Moncharmont B, Tsagris L (1988). Estrogen receptors in cultured rabbit articular chondrocytes: influence of age. J Steroid Biochem.

[B50] Claassen H, Hassenpflug J, Schunke M, Sierralta W, Thole H, Kurz B (2001). Immunohistochemical detection of estrogen receptor alpha in articular chondrocytes from cows, pigs and humans: in situ and in vitro results. Ann Anat.

[B51] Ushiyama T, Ueyama H, Inoue K, Ohkubo I, Hukuda S (1999). Expression of genes for estrogen receptors alpha and beta in human articular chondrocytes. Osteoarthritis Cartilage.

[B52] Richmond RS, Carlson CS, Register TC, Shanker G, Loeser RF (2000). Functional estrogen receptors in adult articular cartilage: estrogen replacement therapy increases chondrocyte synthesis of proteoglycans and insulin-like growth factor binding protein 2. Arthritis Rheum.

[B53] Christiansen C, Tanko LB, Warming L, Moelgaard A, Christgau S, Qvist P (2003). Dose dependent effects on bone resorption and formation of intermittently administered intravenous ibandronate. Osteoporos Int.

[B54] Lehmann HJ, Mouritzen U, Christgau S, Cloos PA, Christiansen C (2002). Effect of bisphosphonates on cartilage turnover assessed with a newly developed assay for collagen type II degradation products. Ann Rheum Dis.

[B55] Cirillo DJ, Wallace RB, Wu L, Yood RA (2006). Effect of hormone therapy on risk of hip and knee joint replacement in the Women's Health Initiative. Arthritis Rheum.

[B56] Oestergaard S, Chouinard L, Doyle N, Karsdal MA, Smith SY, Qvist P (2006). The utility of measuring C-terminal telopeptides of collagen type II (CTX-II) in serum and synovial fluid samples for estimation of articular cartilage status in experimental models of destructive joint diseases. Osteoarthritis Cartilage.

[B57] Zhen EY, Brittain IJ, Laska DA, Mitchell PG, Sumer EU, Karsdal MA (2008). Characterization of metalloprotease cleavage products of human articular cartilage. Arthritis Rheum.

[B58] Meulenbelt I, Kloppenburg M, Kroon HM, Houwing-Duistermaat JJ, Garnero P, Hellio Le Graverand MP (2006). Urinary CTX-II levels are associated with radiographic subtypes of osteoarthritis in hip, knee, hand, and facet joints in subject with familial osteoarthritis at multiple sites: the GARP study. Ann Rheum Dis.

[B59] Meulenbelt I, Kloppenburg M, Kroon HM, Houwing-Duistermaat JJ, Garnero P, Hellio-Le Graverand MP (2007). Clusters of biochemical markers are associated with radiographic subtypes of osteoarthritis (OA) in subject with familial OA at multiple sites. The GARP study. Osteoarthritis Cartilage.

[B60] Dam EB, Byrjalsen I, Karsdal MA, Qvist P, Christiansen C (2008). Increased urinary excretion of C-telopeptides of type II collagen (CTX-II) predicts cartilage loss over 21 months by MRI. Osteoarthritis Cartilage.

